# Leveraging AI-based digital systems in psychological interventions: a research opinion

**DOI:** 10.3389/fdgth.2025.1693504

**Published:** 2025-12-19

**Authors:** Haryasena Panduwiyasa

**Affiliations:** 1 Department of Information System, Telkom University, Bandung, Indonesia; 2 Department of Psychology, School of Psychology, Universitas Insan Cita Indonesia, Jakarta, Indonesia

**Keywords:** digital system, mental health, artificial intelligence, ethical consideration toward AI, psychological intervention

## 1 Introduction

The rapid advancement of digital technology and information systems in the ‘bricks and clicks' era has significantly transformed in various sectors, including mental health services, and professional psychiatric practice (1, 2). As the demand for accessible, efficient, and evidence-based psychological services increases, digital knowledge management systems and technological innovations are being increasingly adopted in mental healthcare (3, 4).

This transformation is not merely technological but represents a paradigm shift in how mental health professionals approach diagnosis, treatment, and professional collaboration (5, 6). The integration of artificial intelligence (AI), telepsychology, and digital knowledge repositories has reshaped the practice of digital mental health intervention (7). These innovations enable practitioners to diagnose conditions more accurately, provide remote therapy, collaborate effectively with multidisciplinary teams, and engage in continuous professional learning (8–10).

However, the widespread adoption of digital tools in mental health also raises several challenges. Ensuring that digital systems adhere to ethical standards while supporting evidence-based practices remains a pressing concern. Issues such as data privacy, algorithmic transparency, clinical accountability, and equitable access must be addressed to maintain patient trust and uphold the integrity of psychological practice (11). Without careful consideration, technological advances risk creating disparities or unintended consequences that undermine their potential benefits (12, 13).

Digital systems open wide range opportunities for interdisciplinary collaboration and the development of innovative therapeutic approaches (14, 15). Through digitalized knowledge management, psychologists can enhance diagnostic accuracy, personalize treatment strategies, and expand the reach of mental healthcare, particularly in underserved or geographically isolated communities where traditional services are limited (16, 17). Such developments highlight the transformative potential of technology to bridge gaps in access and democratize mental health support.

In alignment with the United Nations’ Sustainable Development Goal (SDG) 3, which focuses on promoting well-being and ensuring healthy lives, AI and digital systems can play a crucial role in enhancing the quality, accessibility, and effectiveness of psychological care (18–20). It is imperative to consider both the opportunities and the risks associated with the digital transformation in mental health services. This paper explores the evolving landscape of digital mental health, offering insights into how these technologies can be leveraged to improve care while acknowledging the need for careful ethical and professional consideration (21, 22).

## 2 The role of digital transformation in enhancing mental healthcare

The rapid digital transformation in psychology and mental health services has fundamentally reshaped the landscape of mental healthcare, resulting in an increased dependence on digital knowledge systems to improve service delivery, foster professional collaboration, and achieve better patient outcomes (11, 23). These digitalized knowledge systems provide robust frameworks for the organization, sharing, and utilization of mental health information, thereby facilitating more efficient access to evidence-based practices (3). The convergence of telepsychology, AI-powered decision-making tools, and digital repositories has further revolutionized mental healthcare, creating new opportunities for innovation in the field. A variety of computational and organizational models, including ontological, statistical, behavioral, and hybrid models are now integral to psychological practice, enhancing the scope and precision of interventions (6).

The ontological model of instrumentation plays a crucial role in structuring psychological knowledge, enabling effective representation of clinically relevant information for informed decision-making. Meanwhile, statistical models leverage vast data sets to uncover insights that inform therapeutic approaches, track trends in neurosciences, and predict mental health outcomes (24). Hybrid models, which combine structured knowledge with adaptive learning mechanisms, offer an innovative way to refine psychological interventions continuously. Behavioral models, on the other hand, focus on analyzing user interactions within digital mental health platforms, helping tailor interventions that maximize engagement and efficacy (25).

Despite the clear benefits of digitalized knowledge management in mental healthcare, significant challenges remain. Ensuring the accuracy and reliability of digital knowledge, safeguarding data privacy, and maintaining ethical standards are critical obstacles that need to be addressed (26). As mental health data is highly sensitive, its management must adhere to stringent confidentiality and integrity protocols. Moreover, many mental health professionals face barriers to adopting digital tools, emphasizing the need for further research and development of user-friendly, accessible systems that empower professionals without compromising their ability to deliver high-quality care (6).

Telepsychology has drastically altered the delivery of mental healthcare, enabling remote counselling through video conferencing, mobile applications, and virtual therapy platforms. This transformation has increased access to mental health services, especially for underserved populations (26). Digital knowledge systems are pivotal in telepsychology, enhancing the remote assessment and diagnosis of patients through structured knowledge-sharing. These systems also improve therapist-client interactions by providing digital tools for treatment planning, progress tracking, and real-time feedback. Additionally, AI-driven insights and predictive analytics are empowering clinicians to make data-driven decisions, while secure digital records and knowledge management systems ensure adherence to ethical and professional guidelines (4).

## 3 Psychological ontology in AI-driven decision making

The author(s) declare that Generative AI was used in the creation of this manuscript. According to research by Hee Lee and Yoon (27) integrating Artificial Intelligence (AI) into mental health care services requires the use of a structured ontology to ensure accurate decision-making for psychologists and psychiatrists. The psychological ontology is designed to define and categorize mental conditions, disorders, and treatment responses that are specific to individual patients or clients. By doing so, it enables more accurate diagnoses, allowing AI systems to support clinicians in making data-driven, informed decisions tailored to the unique needs of each patient (28). In utilizing AI-based psychological treatment models, classifying mental disorders according to the Diagnostic and Statistical Manual of Mental Disorders 5 (DSM-5) is essential, as it provides standard diagnostic criteria based on extensive clinical research and expert insights. The synergy between the DSM-5 and AI-based decision-making, including detailed symptomatology analysis, comorbidity patterns, and determinants for disorders such as major depressive disorder, schizophrenia, generalized anxiety disorder, and autism spectrum disorder, enables more precise detection and treatment (29). This approach enables AI models to curate high-fidelity psychiatric data while accounting for inter-individual variability, cultural influences, and differential diagnoses (30). To generate clinically relevant insights, AI-driven systems must integrate structured datasets with multimodal inputs, including patient-reported outcomes, neuroimaging, psychophysiology, and natural language processing (NLP) of clinical narratives. Despite its many benefits, ethical challenges such as algorithmic bias, explainability, and data privacy must be carefully addressed to ensure the safe application of AI in mental healthcare. A robust, scientifically grounded psychological ontology will enable AI to complement mental health professionals by improving diagnostic accuracy, individualized treatment planning, and long-term patient outcomes while upholding applicable clinical and ethical standards in each country.

## 4 Digital knowledge management (DKM) in psychological practice

DKM is an essential framework for enhancing mental healthcare by fostering a knowledge-driven ecosystem that improves clinical decision-making, facilitates research advancements, and optimizes patient outcomes (3, 4). By integrating digital tools, structured knowledge repositories, and artificial intelligence, DKM enables psychologists and mental health professionals to access, share, and apply critical insights in real-time condition. This not only improves diagnostic accuracy and treatment planning but also enhances the efficiency of knowledge dissemination across the mental health community. In the context of mental health, DKM involves more than just storing information, it ensures the seamless integration of digital knowledge systems with therapeutic interventions, supporting evidence-based practices. According to Panduwiyasa et al. (4) DKM consists of three fundamental components which are: People, Process, and Technology. People refer to mental health professionals who share their expertise on digital platforms. Process involves systematic management and dissemination of psychological knowledge through digital repositories. Technology includes digital tools that facilitate the collection, storage, and application of psychological knowledge for decision-making and intervention.

### 4.1 People (psychologists and mental health practitioners)

Mental health professionals, such as psychologists and therapists, are central to the implementation of DKM. Their expertise in diagnosing, treating, and managing mental disorders must be systematically captured, stored, and shared across digital platforms to enhance collective knowledge and ensure consistent, evidence-based care. DKM facilitates collaboration by creating a shared digital space where professionals can engage in peer-to-peer knowledge sharing, participate in digital case studies, and contribute to the development of evidence-based interventions. This collaborative approach not only improves diagnostic accuracy and treatment planning but also encourages continuous professional learning and refinement of clinical practices (31).

### 4.2 Process (clinical knowledge management and decision support)

The process component of DKM in mental health involves a structured system for documenting, retrieving, and disseminating psychological insights, therapeutic methodologies, and clinical research findings. This process begins with collecting data from sources such as patient records, research studies, and therapeutic outcomes, which are then organized in digital repositories. Repositories like the DSM-5 (Diagnostic and Statistical Manual of Mental Disorders) and ICD-11 (International Classification of Diseases) serve as standardized frameworks, ensuring accurate and consistent psychiatric diagnoses and treatment plans by providing clinicians with up-to-date, evidence-based information (32).

The retrieval process enables mental health professionals to quickly access relevant data when making clinical decisions. For example, when encountering a patient, the system can recommend diagnostic criteria based on the patient's symptoms, history, and clinical context. This retrieval is facilitated by search algorithms within the digital system, helping practitioners locate the most relevant and recent guidelines or research. Additionally, the system can identify comorbidities such as depression and anxiety occurring together and suggest treatment options that have been effective for similar cases. These AI-driven recommendations assist clinicians by providing personalized therapy plans, tailored to the patient's data, and aligned with current clinical guidelines (33).

### 4.3 Technology (digital tools for mental health knowledge management)

According to Wang and Lin (30), technology is a fundamental pillar in mental health care, enabling the efficient collection, storage, and utilization of patient data, which helps healthcare providers make well-informed decisions. Among the various advancements, artificial intelligence (AI) plays a crucial role, particularly through its branches such as machine learning (ML) and natural language processing (NLP). ML supports early diagnosis and personalized treatment by detecting patterns in patient data and adapting treatment plans based on patient responses over time. Electronic Health Records (EHRs) further enhance interventions by providing clinicians with seamless access to patient histories, assessments, and progress, ensuring better care coordination and treatment outcomes. NLP aids mental health interventions by analyzing unstructured data, such as patient narratives and therapist notes, to identify psychological patterns and early signs of conditions like depression, anxiety, or PTSD. By processing natural language, NLP provides real-time insights during therapy sessions, enhancing the personalization and responsiveness of care.

AI faces challenges in differentiating between comorbid conditions, which is common in psychological assessment. Disorders like depression and anxiety share overlapping symptoms, making accurate differentiation difficult. AI models can struggle with these nuanced distinctions, leading to potential diagnostic inaccuracies. To improve its diagnostic precision, AI must be trained on diverse, comprehensive datasets and incorporate clinician oversight to validate diagnoses and ensure appropriate treatment. Machine learning supports early diagnosis and personalized treatment by detecting patterns in patient data and adapting treatment plans based on patient responses over time (34). Electronic Health Records (EHRs) further enhance interventions by providing clinicians with seamless access to patient histories, assessments, and progress, ensuring better care coordination and treatment outcomes.

### 4.4 Privacy and data protection

Privacy and data protection are non-negotiable in psychological technology. Given the sensitivity of mental health data, robust security frameworks must be implemented, including end to end encryption, advanced anonymization, fine grained access controls with multi factor authentication, and regular penetration testing. Data storage systems should ensure redundancy and resilience through disaster recovery protocols. Strict compliance with global standards such as General Data Protection Regulation (GDPR) and Health Insurance Portability and Accountability Act (HIPAA) is essential to uphold legal, ethical, and clinical accountability (35).

Crucially, consent management must be transparent, auditable, and patient centered, giving individuals full authority over data access, sharing, and deletion. Emerging technologies like blockchain can significantly strengthen this process by enabling decentralized, tamper proof logs of consent and data transactions, allowing patients to dynamically control their data while ensuring immutable auditability. When used to record metadata or access permissions rather than raw clinical data, blockchain complements traditional safeguards without conflicting with privacy regulations. Continuous monitoring and automated auditing are vital to detect and respond to breaches in real time. Finally, comprehensive training for mental health professionals in cybersecurity, data governance, and ethical digital practices is essential to reduce human error, build patient trust, and ensure the long-term integrity of digital mental health services (36).

## 5 Ethical considerations of AI-use in mental health care

The integration of artificial intelligence (AI) in mental health care presents significant ethical challenges that must be carefully addressed to ensure patient safety, confidentiality, and fairness (37). One of the primary concerns is data privacy and security, as AI-driven mental health applications rely on sensitive personal data, making them vulnerable to breaches and unauthorized access. Ensuring compliance with data protection regulations, GDPR and HIPAA, is essential to maintain patient confidentiality (38). Another ethical issue involves bias and fairness in AI algorithms, as many AI models are trained on datasets that may not fully represent diverse populations. This can lead to disparities in mental health diagnoses and treatment recommendations, disproportionately affecting marginalized communities. Accountability and responsibility are critical considerations, as AI lacks human judgment and may produce erroneous assessments or recommendations (28). The therapeutic relationship is another ethical concern, as AI-based mental health tools, such as chatbots and virtual therapists, may lack the empathy and nuance required for effective psychological support, potentially leading to misinterpretation of emotions or inadequate responses in complex situations (39). Furthermore, informed consent and transparency must be upheld, ensuring that users understand how AI-driven tools function, the limitations of their capabilities, and the extent to which AI influences their mental health care decisions (40).

## 6 The impact of digital systems on mental health interventions

The integration of digital systems, including artificial intelligence (AI), digital knowledge management, and telepsychology, has transformed mental health interventions by increasing accessibility, improving diagnostic precision, and enabling personalized treatment. Machine learning (ML) and natural language processing (NLP) have introduced a data-driven approach to psychological care, allowing for real-time assessments and evidence-based clinical decision-making. AI-powered systems analyze behavioral patterns, linguistic nuances, and physiological markers, facilitating early detection of mental health disorders and the development of tailored intervention strategies. As digital platforms become increasingly embedded in mental health services, these technologies provide scalable and high-impact solutions that help bridge the gap between the growing demand for psychological care and the global shortage of mental health professionals (33). AI-driven decision-making is particularly impactful in mental health diagnosis, where predictive analytics and sentiment analysis can identify symptoms of conditions such as depression, anxiety, and post-traumatic stress disorder (PTSD). These AI models process large datasets derived from e-counseling platforms, chatbot therapy, and telepsychology services, ensuring timely, data-informed psychological interventions. While AI-driven tools are effective, they may lack the nuanced empathy and contextual understanding essential for psychological care (41). The role of digital knowledge management in psychological practice is also crucial in structuring and disseminating evidence-based therapeutic protocols, ensuring uniformity and accuracy in mental health interventions. However, key challenges related to techno-health ethics, AI biases, and encrypted privacy mechanisms must be addressed. AI models are susceptible to biases embedded in training datasets, potentially resulting in inequities in mental health assessments and treatment recommendations (13).

## 7 Conclusion

AI-driven psychological assessments, structured knowledge repositories, and digital mental health interventions have the potential to improve the accuracy, accessibility, and scalability of mental health services. This opinion paper provides a comprehensive overview of the integration of artificial intelligence (AI), digital knowledge management (DKM), and telepsychology in contemporary mental healthcare. Machine learning (ML), natural language processing (NLP), and expert systems have demonstrated effectiveness in automating psychological assessments, personalizing treatment strategies, and supporting traditional therapeutic approaches. Despite these advancements, several challenges continue to limit the full implementation of digital mental health solutions. Ethical considerations related to algorithmic bias, privacy vulnerabilities, and data security risks require robust regulatory frameworks and ongoing oversight. In addition, the limited interpretability of AI models, the central role of empathetic human interaction in therapeutic processes, and potential risks associated with over-reliance on automated decision-making further complicate their adoption. Addressing disparities in digital literacy, mitigating accessibility barriers, and reducing clinician skepticism toward AI-assisted practices are also necessary to ensure equitable and sustainable integration. Future research should prioritize increasing AI transparency, strengthening ethical governance, and fostering collaborative practice models in which AI systems function as supportive tools alongside mental health professionals. The development of a systematic research taxonomy is also recommended to more clearly categorize contextual factors, intervention modalities, and outcome measures, thereby promoting consistency and clarity in the study and application of AI-based digital mental health interventions.
